# Sepsis complicated by brain abscess following ESWL of a caliceal kidney stone: a case report

**DOI:** 10.1590/S1677-5538.IBJU.2015.0727

**Published:** 2016

**Authors:** Alessandro Capitanini, Luca Rosso, Laura Giannecchini, Ophelia Meniconi, Adamasco Cupisti

**Affiliations:** 1 Nephrology Unit, Ospedale di Pescia, Pescia, Italia; 2 Intensive Care Unit, Ospedale di Pescia, Pescia, Italia; 3 Department of Clinical and Experimental Medicine, Università di Pisa, Pisa, Italia

**Keywords:** Lithotripsy, Urolithiasis, Shock, Sepsis, ESWL

## Abstract

A 47-year old, Caucasian man underwent extracorporeal shock wave lithotripsy (ESWL) of a 14mm calcium stone in the right renal pelvis, without urinary tract obstruction or sepsis. 24 hours after ESWL septic shock occurred and the patient was admitted to the Intensive Care Unit (ICU). Escherichia coli emerged from the blood and urine culture. The patient developed acute renal failure and it was necessary to start a continuous renal replacement therapy (CRRT). Infection was successfully treated, patient recovered renal function and an improvement of general condition occurred. The patient was then discharged but three day later the patient returned to the hospital to seek treatment for left facial hemiparesis and hypotonia of his left arm. The brain computed tomography showed a wide abscess (55×75mm) in the frontal right parietal region. A neurosurgical intervention was then performed and the culture of the drained material resulted positive for Escherichia coli. The guidelines of European and American Associations of Urology do not suggest a prophylactic antibiotic therapy for pre-ESWL (except in the presence of risk factors). The serious complication that occurred in the described low risk patient raises the question of whether routine culture and/or antibiotic prophylaxis, is appropriate.

## BACKGROUND

ESWL is a modality of treatment that is widely adopted for renal and ureteral stones. ESWL is generally considered a safe treatment: minor complications are reported in a significant number of patients whereas serious complications, causing ongoing morbidity or mortality, are rare, affecting less than 1% of patients. We describe a serious complication following an uncomplicated kidney stone ESWL in a patient without any symptoms of urinary tract or systemic infections, which resulted in septic shock and metastatic brain abscess.

## CASE PRESENTATION

A 47-year old Caucasian man underwent extracorporeal shock wave lithotripsy (ESWL) for a 14mm calcium-containing stone in the right renal pelvis, without any sign of sepsis or urinary tract obstruction. In the history he reported a renal colic episode and one ESWL treatment about 10 years before. Tonsillectomy, appendicectomy, and history of bronchial asthma with removal of nasal polyps were reported as well.

**Figure 1:**
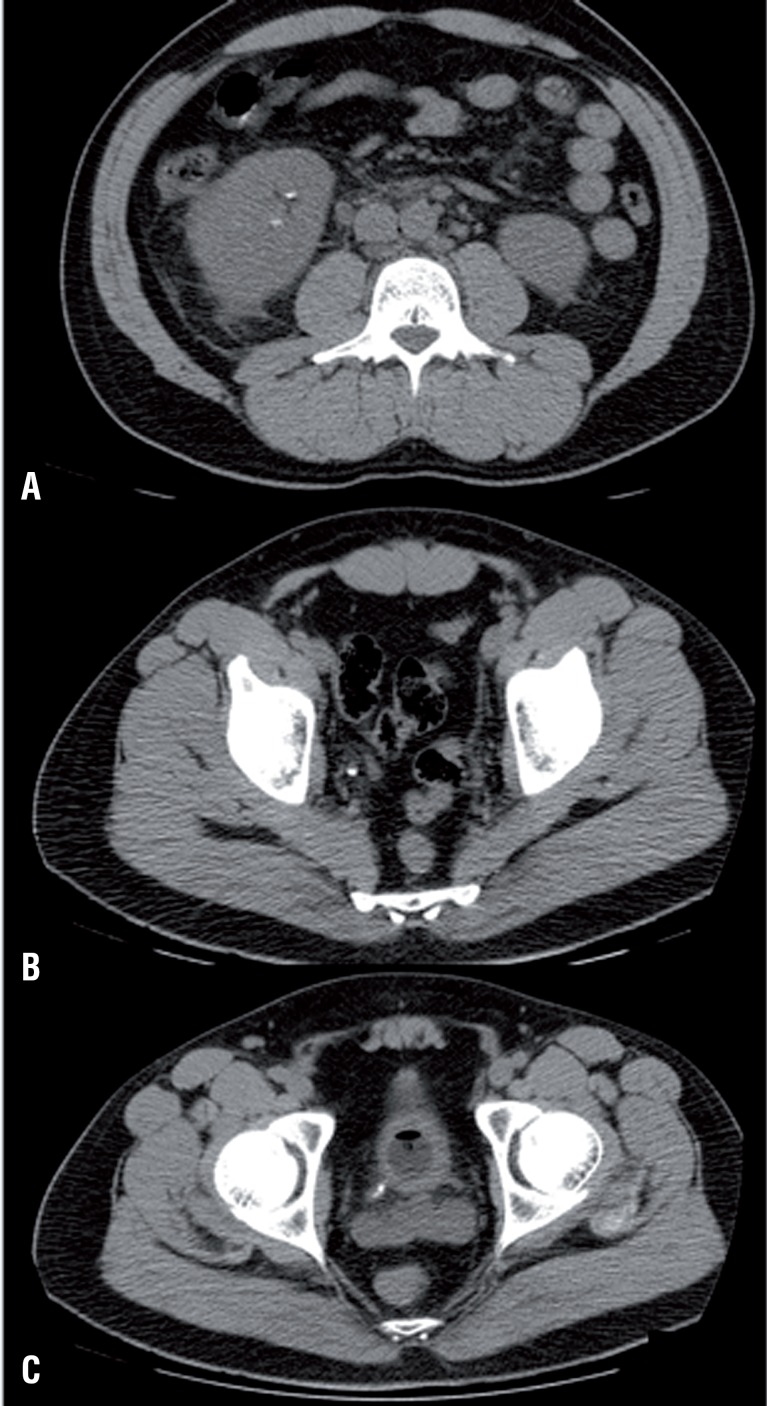
CT at the admission. Residual stone fragments in the lower calcyx (a), and in the lower tract of the ureter (b and c).

**Figure 2:**
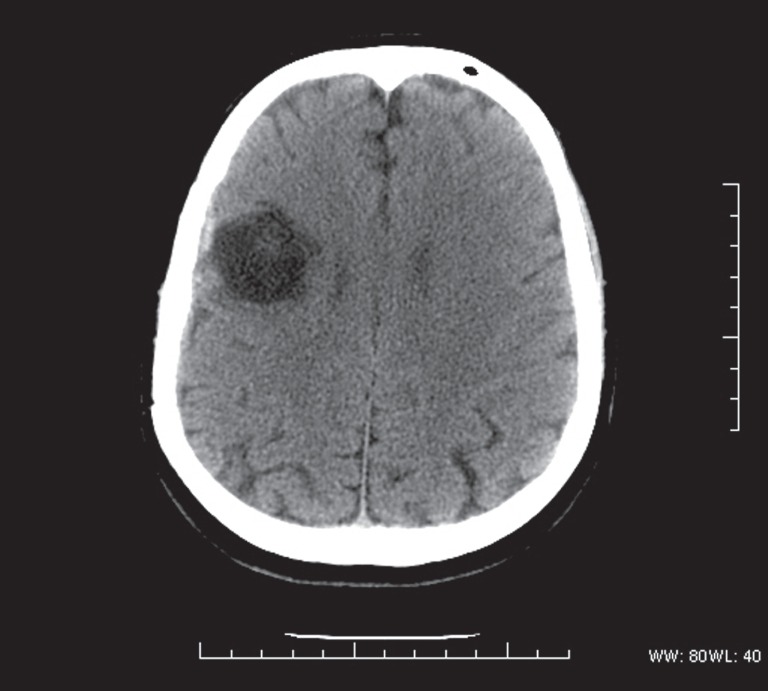
Brain CT showing cerebral abscess of the front-parietal region.

24 hours after the ESWL procedure, the patient experienced malaise, fever, vomiting, and dyspnea, and required hospitalization. The blood gas analysis showed metabolic acidosis with hypoxemia and hypocapnia (pH 7.33, PCO2 31.7mmHg, PO2 49.9mmHg, 84.2% SO2, HCO3 18.1mmol/L, BE-8.1), unstable hemodynamics, arterial blood pressure 90/50mmHg, tachycardia. Laboratory findings showed neutrophilic leukocytosis (WBC 22830/mmc, neutrophils 92.1%), thrombocytopenia (PLT 91.000/mmc), coagulation disorders (41.6% PT, aPTT 55.1″), renal impairment (serum creatinine 4.59mg/dL, BUN 96mg/dL), liver disorders (total bilirubin 2.4mg/dL with direct 1.12mg/ dL, ALT 53U/L, AST 45U/L). CRP was 14.87mg/dL and procalcitonin 48.55ng/mL. The patient showed a gradual onset of respiratory distress that required an oro-tracheal intubation. Chest and abdominal computed tomography showed stone fragments of the right kidney and ureter (Figure-1). The patient was admitted to the ICU with a diagnosis of septic shock after ESWL. The cultures of blood and urine resulted positive for Escherichia coli. The patient started a wide-spectrum antibiotics therapy and continuous renal replacement treatment (CRRT). CRRT was stopped after 72 hours because of the recovery of renal function. Laboratory findings showed an increase of leukocytes (up to 36,230mmc) on the fourth day and then a gradually decrease, while platelets decreased to 8.000mmc on the third day. Liver and kidney biochemical tests gradually returned to the normal range. Patient's general conditions improved, hemo-and urine culture were negative, but, given the persistence of leukocytosis (12,840/mmc), PCR at 7.78mg/dL and fever, it was decided to continue beta-lactam antibiotics (amoxicillin-clavulanate) therapy. The patient was finally discharged but after 3 days he was readmitted because of left facial hemiparesis, hypotonia and loss of strength in the left arm. The brain computed tomography (Figure-2) performed in emergency condition showed a large abscess (55×75mm) in the frontal right parietal region. Neurosurgical intervention was then performed to drain the abscess and the culture of drained fluid resulted positive for Escherichia coli. The patient continued the antibiotic therapy with carbapenems and tigeciclina for 15 days then continued only with carbapenems for a total length of five weeks. Complete neurological recovery occurred.

## DISCUSSION

It's known that urinary tract infections, pyelonephritis and urosepsis may complicate the ESWL procedure. Urinary tract infections occur in about 0.3%, asymptomatic bacteriuria 2.8–5% (1) of cases. Post-ESWL bacteremia occurs in 5% of blood cultures, the development of sepsis after bacteremia is estimated at 1% (2). Pyelonephritis and sepsis occur in 0.3% of patients (3). Among the risk factors identified for the development of post-ESWL infection are larger stone size (>2cm), prior history of urinary tract infections, the presence of calyceal diverticula (4) struvite stones or stent-catheter (3). The existing literature confirms that the occurrence of post-ESWL sepsis is an infrequent condition (5–8). To our knowledge, this is the first report of a central nervous system infection following ESWL of uncomplicated kidney stone, apart from a case of a metastatic endophthalmitis (6). Predisposing conditions cannot always be identified or recognized: for example, an infected stone is not always easily identifiable because not all infected calculi are associated with positivity of urine culture (9). These factors are emphasized even by the American Urology Association (AUA) and the European Urology Association (EUA). Their guidelines agree that, after an extensive literature review and in light of a recent new meta-analysis (10), antibiotic prophylaxis is not necessary for shock wave lithotripsy, especially when no or low risk factors are present. Accordingly, the patient had no indication for prophylactic antibiotic therapy: the stone was 14mm of diameter, located in the renal pelvis, no stents or catheters were present at the moment of the procedure, it was a calcium-containing no-staghorn stone.

## CONCLUSIONS

Notwithstanding AUA and EUA guidelines and recent data from the literature which do not suggest a prophylactic antibiotic therapy preceding-ESWL procedure (except in the presence of known risk factors), in this case urosepsis with metastatic brain abscess occurred following a ESWL treatment of an uncomplicated kidney stone. The meta-analysis (10) on the basis of which the American Urology Association defined its guidelines, recognizes that there is a lack of recent randomized controlled trials that primarily evaluate individual pre-procedure risk factors.

The question arises as to whether a urine culture and/or antibiotic prophylaxis should be routinely considered prior to ESWL treatment of uncomplicated kidney stone.
